# (-)-Epigallocatechin-3-gallate Reduces Cigarette Smoke-Induced Airway Neutrophilic Inflammation and Mucin Hypersecretion in Rats

**DOI:** 10.3389/fphar.2017.00618

**Published:** 2017-09-06

**Authors:** Yingmin Liang, Kenneth W. K. Liu, Sze C. Yeung, Xiang Li, Mary S. M. Ip, Judith C. W. Mak

**Affiliations:** ^1^Department of Medicine, The University of Hong Kong Pokfulam, Hong Kong; ^2^Research Centre of Heart, Brain, Hormone and Healthy Aging, The University of Hong Kong Pokfulam, Hong Kong; ^3^Shenzhen Institute of Research and Innovation, The University of Hong Kong Pokfulam, Hong Kong; ^4^Department of Pharmacology and Pharmacy, The University of Hong Kong Pokfulam, Hong Kong

**Keywords:** cigarette smoke, (-)-epigallocatechin-3-gallate, epidermal growth factor receptor, inflammation, mucus secretion, neutrophil infiltration

## Abstract

**Background:** Cigarette smoking is the leading cause of chronic obstructive pulmonary disease. (-)-Epigallocatechin-3-gallate (EGCG), the major catechins in Chinese green tea, has been studied for its anti-oxidative and anti-inflammatory properties in cell and animal models. In this study, we aimed to analyze the effects of EGCG on cigarette smoke (CS)-induced airway inflammation and mucus secretion in the CS-exposed rat model.

**Methods:** Male Sprague-Dawley rats were randomly divided into either sham air (SA) or CS exposure. EGCG (50 mg/kg b.wt.) was given by oral gavage every other day in both SA and CS-exposed animals. Oxidative stress and inflammatory markers were determined in serum and/or bronchoalveolar lavage fluid by biochemical assays or ELISA. Lung morphological changes were examined by Periodic Acid-Schiff, Masson’s Trichrome staining and immunohistochemical analysis. Western blot analysis was performed to explore the effects of EGCG on epidermal growth factor receptor (EGFR)-mediated signaling pathway.

**Results:** (-)-Epigallocatechin-3-gallate treatment attenuated CS-induced oxidative stress, lung cytokine-induced neutrophil chemoattractant-1 release and neutrophil recruitment. CS exposure caused an increase in the number of goblet cells in line with MUC5AC upregulation, and increased lung collagen deposition, which were alleviated in the presence of EGCG. In addition, CS-induced phosphorylation of EGFR in rat lung was abrogated by EGCG treatment.

**Conclusion:** (-)-Epigallocatechin-3-gallate treatment ameliorated CS-induced oxidative stress and neutrophilic inflammation, as well as airway mucus production and collagen deposition in rats. The present findings suggest that EGCG has a therapeutic effect on chronic airway inflammation and abnormal airway mucus production probably via inhibition of EGFR signaling pathway.

## Introduction

Chronic obstructive Pulmonary Disease (COPD) is a healthcare issue aﬄicting more than 600 million people worldwide, estimated to become the third leading cause of death by the year 2020 (Murray and Lopez, 2013). COPD, which is characterized by the development of irreversible airflow obstruction, is associated with chronic inflammation, leading to progressive decline in lung function (Ito and Barnes, 2009). Exposure to cigarette smoke (CS) is widely attributed to be the major cause of COPD, with accumulation of oxidant burden and airway mucus hypersecretion being pivotal in the pathogenesis of the disease (Macnee, 2007). CS is composed of a complex cocktail of chemicals, able to produce in excess of 10^15^ oxidants per puff (Church and Pryor, 1985). In homeostatic settings, natural antioxidative defenses involving enzymes such as catalases (CAT), superoxide dismutase (SOD), and glutathione s-transferase (GST) would be mounted to combat the oxidative stress elicited by CS exposure (Schieber and Chandel, 2014). Many markers of oxidative stress have been used to describe the effects of cigarette smoking and have been linked to development of COPD including 8-isoprostane, total-antioxidant capacity (T-AOC), and advanced oxidation protein products (AOPP) (Kinnula, 2005; Tavilani et al., 2012; ben Anes et al., 2014). In COPD, mucus hypersecretion has an important influence on small airways, which would further block the existing narrow and obstructed airways, leading to deterioration of lung function. Airway mucus hypersecretion is characterized by goblet cell hyperplasia and metaplasia lining the airway epithelium. Mucin synthesis and secretion in the airways have been reported to be regulated by activation of the epidermal growth factor receptor (EGFR) (Ha and Rogers, 2016). However, there are currently no satisfactory treatments for mucus overproduction, which would help with the alleviation of airway obstruction and the clinical outcomes of COPD.

Chinese green tea consumption has been associated with many beneficial health effects, as we previously demonstrated that Chinese green tea can reduce CS-mediated lung injury in rats (Chan et al., 2009). The beneficial effects of green tea are attributed to the high abundance in phenolic catechins, with the main subtypes being (-)-epigallocatechin-3-gallate (EGCG), (-)-epicatechin-3-gallate (ECG), (-)-epigallocatechin (EGC) and (-)-epicatechin (EC). EGCG is by far the most abundant catechin found in green tea, which can make up to 0.05% of the total content of tea by weight (Higdon and Frei, 2003). Catechins possess strong antioxidant properties, with EGCG and ECG having the highest antioxidant activity, followed by EGC and then EC (Nanjo et al., 1999). Catechins are also thought to possess immunomodulatory properties (Higdon and Frei, 2003). In a mouse smoking model, EGCG partially suppressed BALF inflammatory cells and lactate dehydrogenase activity (March et al., 2006). In airway epithelial cells, EGCG was also found to inhibit release of CS-induced pro-inflammatory cytokines through inhibition of NF-κB activation (Syed et al., 2007). However, there have been few studies regarding the effects of EGCG on mucin 5AC (MUC5AC) overproduction and other pathological alterations such as fibrosis in the airways *in vivo*, and the mechanisms by which EGCG regulate MUC5AC expression have yet to be elucidated.

In the present study, we investigated the effects of EGCG on CS-induced airway inflammation and mucus hypersecretion and the associated signaling pathway using a CS-exposed rat model.

## Materials and Methods

### Materials

Purified EGCG (>95%) was kindly provided by Dr. Hara Yukihiko of Tea Solution, Hara Office Inc. (Tokyo, Japan). Camel cigarettes (with filter, 11 mg tar, 0.8 mg nicotine) were purchased commercially (R.J. Reynolds, Winston-Salem, NC, United States). The bioassay kits of total anti-oxidant capacity (T-AOC), enzyme activity of total superoxide dismutase (SOD) and glutathione s-transferase (GST) were purchased from Nanjing Jiancheng Bioengineering Institute (Nanjing, Jiangsu, China) and enzyme activity of catalase (CAT) was from Molecular Probes Inc., Invitrogen (Eugene, OR, United States). The enzyme-linked immunosorbent assay (ELISA) kits for 8-isoprostane, cytokine-induced neutrophil chemoattractant-1 (CINC-1, resembles to human IL-8) and monocyte chemotactic protein-1 (MCP-1, CCL2) were purchased from Cayman (Ann Arbor, MI, United States), R&D systems (Minneapolis, MN, United States) and BD Biosciences (San Jose, CA, United States) respectively. Periodic Acid-Schiff (PAS) and Masson’s Trichrome staining systems were purchased from Sigma–Aldrich Co. LLC (St. Louis, MO, United States).

### Animal Treatment

Male Sprague-Dawley rats (220–260 g) were exposed to cigarette smoke using a home-made system previously established by our group (Chan et al., 2009; Lau et al., 2012). Animals were placed in a 20-liter Perspex chamber, under a constant stream of CS (4% v/v, smoke/air) passed through the chamber for 1 h daily, for 56 consecutive days. Rats were randomly divided into four groups: sham air with water (SA group), cigarette smoke exposure with water (CS group), sham air with EGCG (50 mg/kg body weight) by oral gavage every other day (EGCG/SA group), and cigarette smoke exposure with EGCG (50 mg/kg body weight) by oral gavage every other day (EGCG/CS group). The dose of EGCG used in this study was chosen based on previously published data (Chan et al., 2010). All studies were approved by the Committee on the Use of Live Animals in Teaching and Research (CULATR, No. 2702-12) at the University of Hong Kong. All animals were purchased from the Laboratory Animal Unit at the University of Hong Kong. Animals were housed in a controlled environment (22 ± 1°C), humidity (65–70%) on a 12/12-h light/dark cycle.

Approximately 24 h after the last CS exposure, the body weight of rats was measured and the animals were euthanized with lethal dose (100 mg/kg body weight) of phenobarbital for blood and tissue harvesting. An incision was made at the upper part of the trachea and bronchoalveolar lavage (BAL) was collected by instilling lungs with 1.5 ml ice-cold phosphate buffer saline (PBS) for three times in total. The BAL was centrifuged at 3,000 rpm for 15 min and the supernatant was collected as bronchoalveolar lavage fluid (BALF). Serum was separated by centrifugation at 1,500 *g* for 10 min. Serum, BALF and lung tissues were stored at -80°C until use.

### Histological and Morphometric Analyses

The largest lobe of the left lung tissue was fixed in 4% formalin solution and embedded in paraffin (Chan et al., 2009). Paraffin-embedded blocks of lung tissues were cut to 5-μm thickness using a microtome, and the deparaffinized tissue sections were subjected to PAS staining to identify goblet cells or Masson’s Trichrome to identify collagen deposition. Images of 5 fields for epithelium in cartilaginous bronchus were captured randomly at ×20 magnifications by using a Nikon Eclipse Ni (Nikon Instruments Inc., Tokyo, Japan) with a SPOT RT3 camera (Model 25.4 2 Mp Slider, SPOT Imaging Solutions, Sterling Heights, MI, United States). The PAS-positive areas were measured using the SPOT Software 5.0. Quantification of the Masson’s Trichrome staining was analyzed by Image J (NIH) with additional threshold color plug-ins to process the file images in tiff. Pixels corresponding to the area stained in blue for Masson’s Trichrome staining were normalized to the total pixel area of the tissue image and the results were expressed as positive area (%). Images were analyzed by two independent and blinded investigators.

### Measurement of Oxidative Stress and Inflammatory Markers

Serum AOPP level was determined as previously described (Lau et al., 2012). AOPP level is expressed in μM chloramine-T equivalents. Serum level 8-isoprostane and activities of T-AOC, SOD, CAT and GST were assayed by commercially available kits. The measurement of T-AOC was based on the ferric reducing/antioxidant power (FRAP) method as previously described (Qiao et al., 2006). One unit of T-AOC is defined as the OD value of the reaction system increase 0.01 per minute at 37°C. One unit of total SOD activity is defined as the amount of enzyme needed to exhibit 50% dismutation of the superoxide radical in 1 ml reaction buffer. One unit of CAT activity is defined as the amount of enzyme that will decompose 1 μmole of H_2_O_2_ per minute at pH 7.0 at 25°C. One unit of GST activity is defined as 1 μmole of GSH decreased per minute under the conditions of 37°C. Serum and BALF levels of CINC-1 and MCP-1 were determined with commercially available kits.

### Western Blot Analysis

Frozen lung tissues were homogenized using mortar and pestle as previously described (Chan et al., 2009). Supernatants were assayed for protein concentration by Bradford method using bovine serum albumin as standard. Lung proteins (40 μg) were separated on 10% SDS–PAGE and then transferred onto a nitrocellulose membrane. After blocking in 5% non-fat milk, membranes were incubated with diluted specific primary antibodies (p-EGFR: #YP0526, EGFR: #YT1485, ImmunoWay Biotechnology Company, Plano, TX, United States; β-actin: #4970, Cell Signaling Technology Inc., Danvers, MA, United States) overnight at 4°C. Then the membranes were incubated with horseradish peroxidase (HRP)-conjugated goat-anti-rabbit IgG secondary antibodies (Novus Biologicals, Littleton, CO, United States) and visualized by medical X-ray film using enhanced chemiluminescence (ECL; Amersham Biosciences, Piscataway, United Kingdom). Densitometry analysis of the bands was performed using ImageJ.

### Immunohistochemistry and Immunohistofluorescent Staining

The deparaffinized and hydrated lung section was placed in a 10 mM citric acid solution (pH6.0) and incubated at 120°C for 10 min. Sections were cooled to room temperature and then washed with PBS for 10 min. After blocking with 10% house serum in antibody diluent for 1 h, sections were stained with the primary antibody (MUC5AC) overnight at 4°C and then incubated with ImmPRESS^TM^ Universal Reagent anti-mouse/rabbit IgG peroxidase (Vector Laboratories, Burlingame, CA, United States) for 1 h. Diaminobenzidine (DAB) (Pierce Biotechnology, Thermo Fisher Scientific, Waltham, MA, United States) was prepared according to the manufacturer, and 100 μl of solution was added to each section for 3–5 min. Sections were then rinsed in distilled water and underwent conventional dehydration and then mounted. The extent of MUC5AC-positive staining in each airway was analyzed following the same method as PAS staining.

After rehydration and blocking as described above, lung sections were stained with primary antibody (neutrophil elastase, NE, ab21595; Abcam, Cambridge, MA, United States) overnight at 4°C and then incubated with fluorescence-labeled secondary antibody and mounting medium containing DAPI (Invitrogen). The imaging analysis was performed by counting number of positive cells and the total amount of nuclei in each field. The ratio of positive cells to the total cell number is calculated to avoid the effect of cell density in lung sections.

### Statistical Analysis

Results are expressed as means ± SEM. For comparisons between groups, analysis of variance (ANOVA) with Bonferroni *post hoc* analysis was used. All analyses were performed using Prism (Version 6.0, GraphPad, San Diego, CA, United States), with a value of *p* < 0.05 regarded as significant.

## Results

### Effects of EGCG on Cigarette Smoke (CS)-Induced Oxidative Stress

**FIGURE 1:**
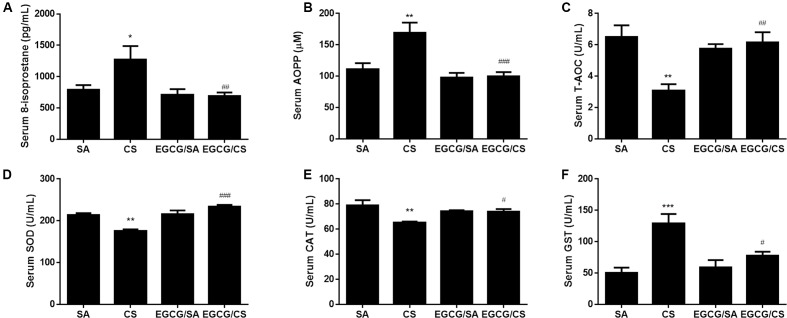
Effect of EGCG on serum oxidative stress markers in rats. 8-isoprostane level **(A)** and advanced oxidation protein products (AOPP) level **(B)** were significantly increased in CS-exposed group of rats compared to SA group. Oral administration of EGCG significantly decreased 8-isoprostane and AOPP levels compared to CS group. Total antioxidant capacity (T-AOC) **(C)**, total superoxide dismutase (SOD) activity **(D)**, and catalase (CAT) activity **(E)** were inhibited in CS-exposed rats while EGCG treatment reversed these enzymes activities. CS exposure upregulated the activity of glutathione s-transferase (GST) and EGCG treatment reversed GST activity **(F)**. The results are expressed as means ± SEM; *n* = 7 each group. SA group, water/sham air; CS group, water/cigarette smoke; EGCG/SA group, EGCG (50 mg/kg)/sham air; EGCG/CS, EGCG (50 mg/kg)/cigarette smoke. ^∗^*p* < 0.05, ^∗∗^*p* < 0.01 and ^∗∗∗^*p* < 0.001 for the comparison between CS and SA group, ^#^*p* < 0.05, ^##^*p* < 0.01 and ^###^*p* < 0.001 for the comparison between EGCG/CS and CS group.

Levels of oxidative stress markers, including 8-isoprostane and AOPP, were elevated by CS in serum compared to SA group (*p* < 0.05 and *p* < 0.01 for CS and SA groups, respectively). EGCG treatment reversed CS-induced elevation of serum levels of 8-isoprostane and AOPP to SA group (*p* < 0.01 and *p* < 0.001, respectively). EGCG treatment alone had no effect on serum levels of 8-isoprostane and AOPP levels (**Figures 1A,B**).

Cigarette smoke exposure reduced the serum T-AOC and the activities of antioxidant enzymes such as SOD and CAT (*p* < 0.01 for CS and SA groups, respectively), which was blocked by EGCG administration (T-AOC: *p* < 0.01; SOD: *p* < 0.001; and CAT: *p* < 0.05) (**Figures 1C–E**).

Serum GST activity was elevated to more than twofolds after CS exposure (*p* < 0.001 for CS and SA groups, respectively), which was inhibited in the presence of EGCG (*p* < 0.05) (**Figure 1F**).

### Effects of EGCG on CS-Induced Lung Inflammation

**FIGURE 2:**
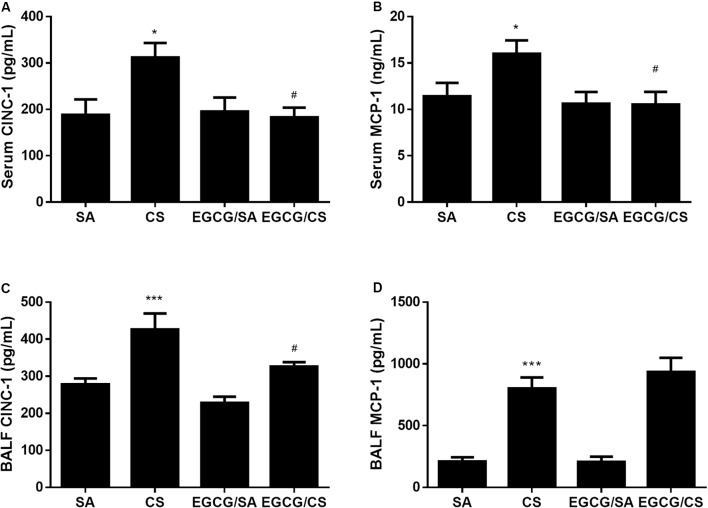
Effects of EGCG on CINC-1 and MCP-1 levels in serum and BALF of rats. Both CINC-1 and MCP-1 levels in serum and BALF were significantly elevated in the rats exposed to CS for 56 days **(A–D)**. EGCG attenuated CS-induced CINC-1 elevation in serum **(A)** and BALF **(C)** and reduced serum MCP-1 level **(B)** but not BALF MCP-1 level **(D)**. The results are expressed as means ± SEM; *n* = 7 each group. SA group, water/sham air; CS group, water/cigarette smoke; EGCG/SA group, EGCG (50 mg/kg)/sham air; EGCG/CS, EGCG (50 mg/kg)/cigarette smoke. ^∗^*p* < 0.05 and ^∗∗∗^*p* < 0.001 for the comparison between CS and SA group, ^#^*p* < 0.05 for the comparison between EGCG/CS and CS group.

Cigarette smoke elevated levels of inflammatory chemokines CINC-1 and MCP-1 (**Figure 2**), in serum and BALF (*p* < 0.05 for serum CINC-1 and MCP-1, *p* < 0.001 for BALF CINC-1 and MCP-1 for CS and SA groups, respectively). EGCG attenuated the CS-induced elevation of CINC-1 in both serum and BALF (*p* < 0.05, respectively), while EGCG attenuated the CS-induced elevation of MCP-1 in serum (*p* < 0.05) but not in BALF (*p* > 0.05).

### Effect of EGCG on CS-Induced Neutrophil Infiltration in Rat Lung

**FIGURE 3:**
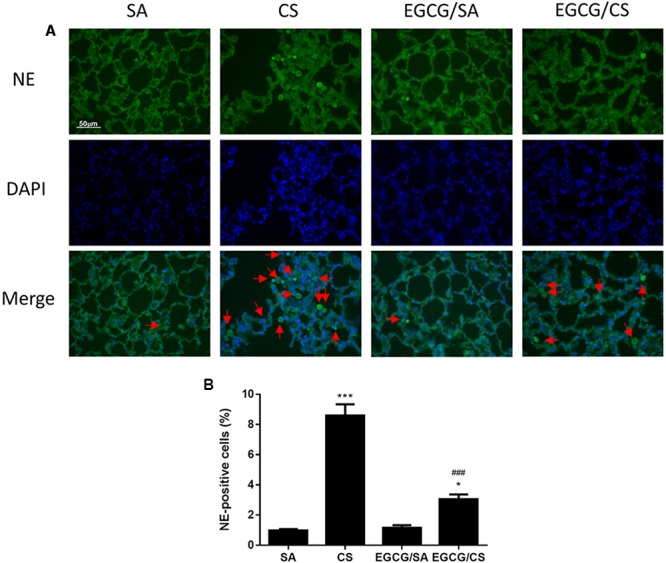
Effect of EGCG on neutrophil infiltration in rat lung. Rats treated with EGCG or control vehicle were killed on day 57 and the left lung was formalin-fixed and sectioned for immunofluorescence (magnification ×400). **(A)** Representative immunofluorescence images of tissue neutrophils as neutrophil elastase (NE)-positive cells with green fluorescence in lung sections. Nuclei were stained with DAPI in blue fluorescence. Scale bar, 50 μm. *Arrows* indicate representative cells with positive staining. **(B)** Quantification of NE-positive cells in the lung sections. The results are expressed as means ± SEM; *n* = 6 each group. SA group, water/sham air; CS group, water/cigarette smoke; EGCG/SA group, EGCG (50 mg/kg)/sham air; EGCG/CS, EGCG (50 mg/kg)/cigarette smoke. ^∗^*p* < 0.05 and ^∗∗∗^*p* < 0.001 for the comparison to SA group, ^###^*p* < 0.001 for the comparison between EGCG/CS and CS group.

To examine the infiltration of neutrophils in the lung, lung sections were immunostained with NE (**Figure 3A**). CS exposure increased the number of infiltrated neutrophil as NE-positive cells in the lung (*p* < 0.001 for comparison between CS and SA groups). EGCG treatment reduced the number of neutrophils significantly (*p* < 0.001) (**Figure 3B**).

### Effect of EGCG on CS-Induced Mucus Hypersecretion

**FIGURE 4:**
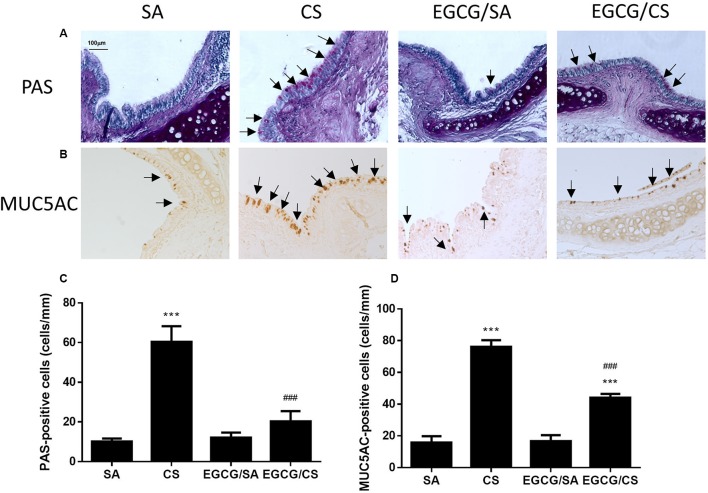
Effect of EGCG on airway mucus secretion in rats. Rats treated with EGCG or control vehicle were killed on day 57 and the left lung was formalin-fixed and sectioned for histology/immunohistochemistry (magnification ×200). **(A)** Representative photomicrographs of lung sections stained with periodic acid Schiff (PAS). Goblet cells appear as purple staining (*arrows*) over epithelium. PAS staining revealed increased goblet cell metaplasia after CS exposure and EGCG reduced the CS-induced goblet cell metaplasia. **(B)** Immunohistochemistry for MUC5AC was performed using an anti-MUC5AC peptide mouse polyclonal antibody, and detected with an anti-mouse/rabbit IgG peroxidase antibody and diaminobenzidine (DAB). MUC5AC-positive staining showed similar phenomenon of mucus secretion of PAS-staining in different groups. Scale bar, 100 μm. *Arrows* indicate representative cells with positive staining. **(C)** Quantification of PAS-positive cells per length of epithelium for goblet cells of different groups. **(D)** Quantification of MUC5AC-positive cells per length of epithelium for mucin of different groups. The results are expressed as means ± SEM; *n* = 5–6 each group. SA group, water/sham air; CS group, water/cigarette smoke; EGCG/SA group, EGCG (50 mg/kg)/sham air; EGCG/CS, EGCG (50 mg/kg)/cigarette smoke. ^∗∗∗^*p* < 0.001 for the comparison to SA group, ^###^*p* < 0.001 for the comparison between EGCG/CS and CS group.

To confirm the effect of EGCG on airway mucus hypersecretion in CS-exposed rat, the lung section was stained with PAS (**Figure 4A**). CS exposure significantly caused upregulation of the number of goblet cells containing mucus in the epithelial surface of cartilaginous bronchus (*p* < 0.001). Treatment with EGCG significantly reduced the number of goblet cells (*p* < 0.001) (**Figure 4C**).

MUC5AC is recognized as the major secreted airway mucins, which is highly expressed in the goblet cells of the airway epithelium. Immunohistochemical experiment showed that the MUC5AC-positive cells were co-localized in PAS-stained goblet cells (**Figure 4B**). The MUC5AC-positive staining in airway epithelium was increased in CS-exposed rat (*p* < 0.001), which was abolished by EGCG administration (*p* < 0.001) (**Figure 4D**). Therefore, EGCG could inhibit CS-induced mucus hypersecretion in the airways of rats.

### Effect of EGCG on CS-Induced Lung Fibrosis

**FIGURE 5:**
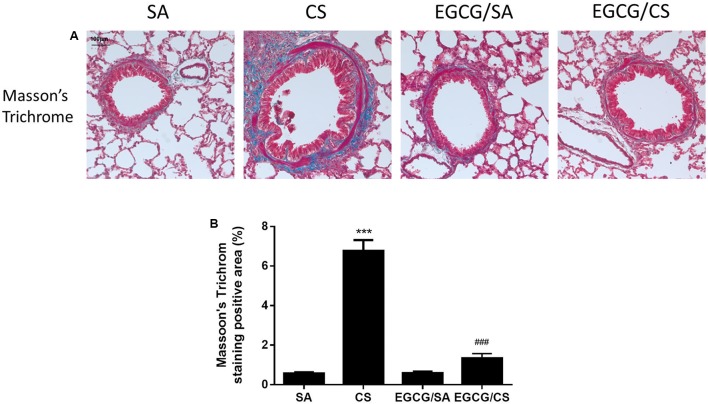
Effect of EGCG on airway fibrosis in rats. Rats treated with EGCG or control vehicle were killed on day 57 and the left lung was formalin-fixed and sectioned for histology (magnification ×200). **(A)** Representative photomicrographs of lung sections stained with Masson’s Trichrome staining system. More collagen deposition/fibrosis in or near the airway wall was found in CS group compared to SA group. Compared with the CS group, collagen deposition in the EGCG/CS group was significantly reduced. Scale bar, 100 μm. **(B)** Quantification of staining-positive area (%) of the lung sections from different group. The results are expressed as means ± SEM; *n* = 5–6 each group. SA group, water/sham air; CS group, water/cigarette smoke; EGCG/SA group, EGCG (50 mg/kg)/sham air; EGCG/CS, EGCG (50 mg/kg)/cigarette smoke. ^∗∗∗^*p* < 0.001 for the comparison between CS and SA group, ^###^*p* < 0.001 for the comparison between EGCG/CS and CS group.

Small airway remodeling is a common feature of COPD patients. The sub-epithelial deposition of collagen in airways of medium size indicates fibrosis. Masson’s Trichrome staining was used to evaluate the presence and the distribution of the collagen in the lung (**Figure 5A**). CS increased collagen deposition in and around the airway wall in the lungs compared to SA group (*p* < 0.001). In the EGCG/CS group, the collagen deposition significantly reduced compared to CS group (*p* < 0.001) (**Figure 5B**).

### Involvement of EGFR in EGCG Action against CS-Induced Mucus Secretion

**FIGURE 6:**
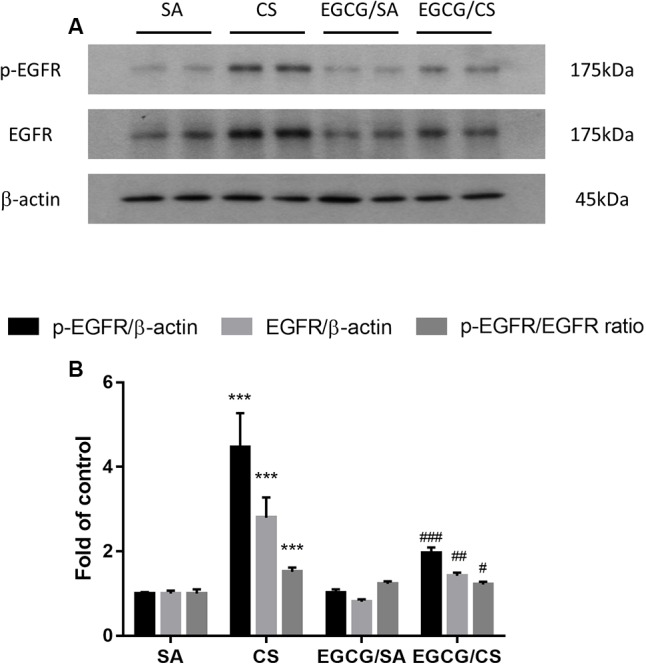
Effect of EGCG on the activation of EGFR in rats. **(A)** Representative photographs of Western blot for p-EGFR and EGFR proteins from homogenized rat lung tissue were shown. **(B)** Protein levels were normalized to the corresponding β-actin and the ratios of p-EGFR/EGFR were calculated. Western blot analysis showed that EGCG suppressed CS-induced elevated phosphorylation of EGFR, total EGFR and the ratio of p-EGFR/EGFR. The results are expressed as means ± SEM; *n* = 7 each group. SA group, water/sham air; CS group, water/cigarette smoke; EGCG/SA group, EGCG (50 mg/kg)/sham air; EGCG/CS, EGCG (50 mg/kg)/cigarette smoke. ^∗∗∗^*p* < 0.001 for the comparison between CS and SA group, ^#^*p* < 0.05, ^##^*p* < 0.01, ^###^*p* < 0.001 for the comparison between EGCG/CS and CS group. EGFR, epidermal growth factor receptor.

To further explore the mechanism how EGCG ameliorated CS-induced mucus hypersecretion, the changes of protein expression of p-EGFR and EGFR were detected using Western blot analysis. CS exposure upregulated the expressions of p-EGFR and EGFR by 4.5- and 2.8-fold, respectively, and increased the ratio of p-EGFR/EGFR significantly, which were inhibited in the presence of EGCG treatment to the same extent (**Figure 6** and Supplementary Figure S1).

## Discussion

In this study, we demonstrated that the imbalance of serum levels of oxidants and antioxidants by CS exposure was reversed by EGCG administration, in support of EGCG being an antioxidant. We further found that EGCG inhibited CS-induced goblet cell hyperplasia and reduced number of MUC5AC-positive cells in the airways, CINC-1 production, neutrophil infiltration and collagen deposition probably via the inhibition of EGFR activation in rat lung. To the best of our knowledge, this is the first study to reveal the signaling pathway involved in EGCG-mediated inhibition of mucus hypersecretion *in vivo*.

In contrast to our previous study, Chinese green tea (Lung Chen) caused body weight loss (Chan et al., 2009) but not EGCG treatment. At baseline, the body weight was similar among all groups. By week 8, the weight gain in the CS group was slightly lower than in the SA group (data not shown). Therefore, the loss of body weight from green tea should be due to the presence of the combination of catechins rather than EGCG alone.

In the current study, serum 8-isoprostane, a biomarker of oxidative stress, was increased after CS exposure in agreement with COPD patients and animal studies (Chan et al., 2009; Kazmierczak et al., 2015). Another oxidative stress biomarker AOPP, as a measure of protein oxidation caused by the chlorinated oxidants, was associated with the status of smoking in humans (Vargas et al., 2013), in agreement with our current findings. In response to the increase in oxidant load, we found a decrease in serum T-AOC, and a reduction in activities of both SOD and catalase upon CS exposure. In agreement, T-AOC in saliva was reduced in smokers compared with non-smokers (Bakhtiari et al., 2015), and the activities of SOD and catalase, the most important antioxidant enzymes, were reduced in serum of smokers and COPD patients compared to normal controls (Tavilani et al., 2012). GSTs, phase II enzymes that catalyze the conjugation of electrophilic molecules with glutathione (GSH) to detoxified conjugates were increased in mild COPD patients but reduced in severe COPD patients (Harju et al., 2008) compared to normal controls. In this study, we found an increase in serum GST levels after CS exposure, indicating that our animal model mimics milder stages of human COPD. Similar to our previous findings with Chinese green tea (Lung Chen), we found that the pure compound EGCG could effectively reverse CS-induced imbalance on oxidants/antioxidants, supporting the role of green tea catechin EGCG in controlling oxidative stress.

Smoking is regarded as one of major risk factors in chronic inflammatory lung diseases. Components of CS have strong oxidative effects and are believed to drive the downstream inflammatory processes, especially in the small airways (Hogg, 2004). A variety of inflammatory and immune cells, including macrophages, neutrophils, CD8+ lymphocytes, and their release of multiple inflammatory mediators were involved in such process (Barnes, 2004). Our previous study also demonstrated that CS caused infiltrations of macrophages and neutrophils in the airways (Li et al., 2017). In this study, we focused on CINC-1 (resemble to human IL-8) and MCP-1, which regulate the recruitment and maturation of neutrophils and macrophages, respectively. Higher gene expression levels of IL-8 and MCP-1 were found in the bronchiolar epithelium of subjects with COPD (de Boer et al., 2000) and increased levels of IL-8 in sputum and BALF of patients with COPD compared with healthy smokers (Keatings et al., 1996; Tanino et al., 2002). Upon CS exposure, both CINC-1 and MCP-1 were upregulated in serum and BALF. Interestingly, EGCG administration reduced the levels of CINC-1 in both serum and BALF, but not MCP-1 in BALF (**Figure 2**), suggesting the presence of differential effects. The exact mechanism in which EGCG mediates such an effect may be due to direct binding to the chemokines and limiting their biological activities (Qin et al., 2011). The mechanism for the differential effects between serum and BALF MCP-1 was currently unclear. In this study, EGCG was administered via oral gavage with its active ingredients absorbed into the blood stream. Neutrophils are recruited into the lung from the circulation via CINC-1 as chemoattractant. On the other hand, there are two types of macrophages found in the lung, i.e., resident alveolar macrophages and macrophages differentiated from circulating monocytes. MCP-1 plays a critical role in regulating the activation and recruitment of cells of the monocytic lineage. Therefore, the predominant resident alveolar macrophages may not be an essential target of EGCG via oral gavage to detect an inhibitory effect on MCP-1 in BALF. Although there was no inhibition of CS-induced elevation of MCP-1 in BALF, EGCG significantly inhibited CS-mediated recruitment of neutrophils, in line with the inhibition of CINC-1 in both serum and BALF.

Mucus is essential because of its role in protecting the airways. However, chronic inflammatory lung diseases, such as COPD, are often associated with excessive mucus production, especially in cases of chronic bronchitis. Cigarette smoke is a common stimulus that promotes mucus secretion and has a great effect on promoting the synthesis and secretion of MUC5AC mucin (Haswell et al., 2010). MUC5AC has been recognized as the predominant mucin in human airway epithelial cells, and its expression increases in smokers and patients with COPD (Di et al., 2012). In this study, CS exposure caused increased numbers of goblet cells and the MUC5AC-positive cells in the airways of rats as an indicator for the production of more mucus due to oxidative stress. In agreement, oxidants were found to be involved in mucus hypersecretion and impairment of mucociliary clearance (Fischer et al., 2015). Hydrogen peroxide and superoxide were shown to cause increased mucus secretion in airway epithelial cells (Adler et al., 1990). Our data revealed that EGCG reversed the activities of SOD and CAT, which could reduce the levels of hydrogen peroxide and superoxide, resulting in attenuation of mucus production, which may suggest the partial attribution of antioxidative effect of EGCG on mucus secretion. On the other hand, excessive neutrophilia and neutrophil chemoattractants, like IL-8 and CINC-1/GRO-α, have been linked to the development of the mucus hypersecretory phenotype present in COPD patients (Nadel, 2000). Neutrophils are the predominant inflammatory cells in the airways of patients with COPD. Neutrophilic products, such as proteases and oxidants, have been evaluated for their roles in regulating mucin gene expression. NE, a serine protease, is regarded as a major product from activated neutrophils to be implicated in airway inflammation, goblet cell metaplasia, structural lung damage, and host defense (Gehrig et al., 2014). NE, which can then act directly on the epithelium (Takeyama et al., 2000), increases MUC5AC gene expression by inducing oxidative stress (Fischer and Voynow, 2000) or releasing transforming growth factor (TGF)-α, which results in EGFR activation (Kohri et al., 2002). In the present study, the neutrophil counts were elevated in CS-exposed rats and significantly lowered in EGCG-pretreated rats. Therefore, the inhibitory effect of EGCG on mucus hypersecretion may be partly due to the inhibition of neutrophil infiltration.

The EGFR signaling pathway is a receptor tyrosine kinase and plays a regulatory role in airway mucus production and secretion (Ha and Rogers, 2016). Takeyama et al. (2000) found that ligand-independent EGFR phosphorylation occurred in response to oxidative stress induced by activated neutrophils. Furthermore, Takeyama demonstrated that CS exposure upregulated EGFR mRNA expression and induced EGFR-specific tyrosine phosphorylation, resulting in increased MUC5AC mRNA and protein production, such effects that were inhibited completely by selective EGFR tyrosine kinase inhibitors (Takeyama et al., 2001). In this study, CS increased the neutrophil infiltration in the airways, increased p-EGFR and EGFR protein levels in rat lung tissue, resulting in increased MUC5AC production, which was inhibited in the presence of EGCG, indicating the protective effect on CS-mediated airway mucus hypersecretory diseases through the EGFR pathway. In support of our findings, the theaflavins isolated from black tea and ECG extracted from green tea have been reported to inhibit mucin secretion possible via EGFR pathway (Kim et al., 2010; Wu et al., 2012).

Pulmonary fibrosis is a hallmark of repeated cycles of dysregulated repair and remodeling of the lung parenchyma, causing decreased airway elasticity. The role of fibrosis in COPD is somewhat controversial but recent evidence suggests that fibrosis and COPD do co-exist, usually in smokers (Jankowich and Rounds, 2012). Collagen fibers, being one of the main components of the extracellular matrix, undergo constant remodeling upon injury caused by exposure to CS (McKleroy et al., 2013). Increased collagen deposition was found to correlate with lung destruction in human emphysema (Martin-Mosquero et al., 2006). In this study, rats exposed to CS increased collagen deposition in the lung, which was attenuated by EGCG treatment. The exact mechanisms involved are not entirely clear, although some studies have inferred that catechins are potentiating stabilization of collagen fibers as well as the inhibition of collagenases (Madhan et al., 2005, 2007).

## Conclusion

**FIGURE 7:**
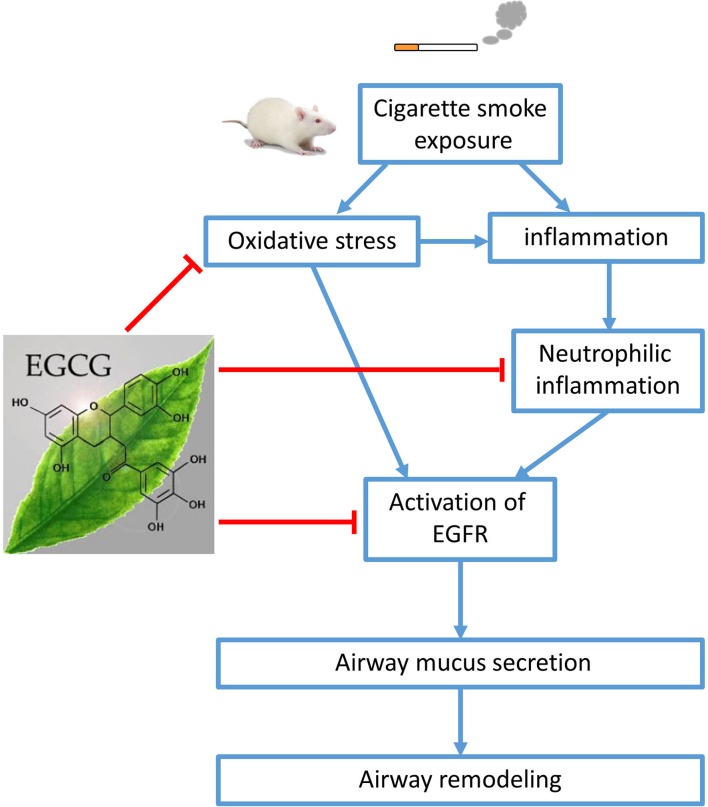
Schematic diagram. Cigarette smoke (CS) exposure causes oxidative stress, inflammation, and airway mucus production. (-)-Epigallocatechin-3-gallate (EGCG) attenuated CS-induced oxidative stress, neutrophilic airway inflammation, and airway mucus production in rat lungs probably via the inhibition of epidermal growth factor receptor (EGFR) signaling pathway, leading to amelioration of airway remodeling.

Our data demonstrate that EGCG has a protective effect on CS-induced oxidative stress, airway neutrophilic inflammation, and airway mucus production in rat lungs probably via inhibition of EGFR signaling pathway, leading to amelioration of airway remodeling (**Figure 7**). EGCG may be a promising therapeutic strategy to limit neutrophil recruitment and to treat mucus hypersecretion in the airways of smokers without or with COPD.

## Ethics Statement

This article does not contain any studies with human participants performed by any of the authors. All procedures performed in studies involving animals were in accordance with the ethical standards of the Committee on the Use of Live Animal in Teaching and Research (CULATR) in the University of Hong Kong with the approval number 2702-12.

## Author Contributions

YL conceived, performed and interpreted the experiments and wrote the draft of the manuscript. KL helped with experimental design and writing the draft of the manuscript. SY helped with the animal study and section staining, and analyzed the data. XL helped with the animal study and sample preparation. MI helped with the experimental design and editing the manuscript. JM conceived, interpreted the experiments and assisted with editing the manuscript. All authors reviewed and approved the manuscript.

## Conflict of Interest Statement

The authors declare that the research was conducted in the absence of any commercial or financial relationships that could be construed as a potential conflict of interest.
