# Supplementary material for: (-)-Epigallocatechin-3-gallate Reduces Cigarette Smoke-Induced Airway Neutrophilic Inflammation and Mucin Hypersecretion in Rats
Source: Front Pharmacol. 2017 Sep 6;8:618. doi: 10.3389/fphar.2017.00618 (PMC5592236; doi:10.3389/fphar.2017.00618)
Supplement: Supplementary file 1 [file Data_Sheet_1.PDF]

Supplementary Material

(-)-Epigallocatechin-3-gallate reduces cigarette smoke-induced airway neutrophilic inflammation and mucin hypersecretion in rats

EGCG attenuates smoking-induced lung injury

Yingmin Liang, Kenneth W. K. Liu, Sze Chun Yeung, Xiang Li, Mary S. M. Ip, Judith C. W. Mak

SUPPLEMENTAL RESULT(S)

EGFR signaling pathway

Full images of Western blot of p-EGFR, EGFR, and β-actin were shown in the Figure S1.

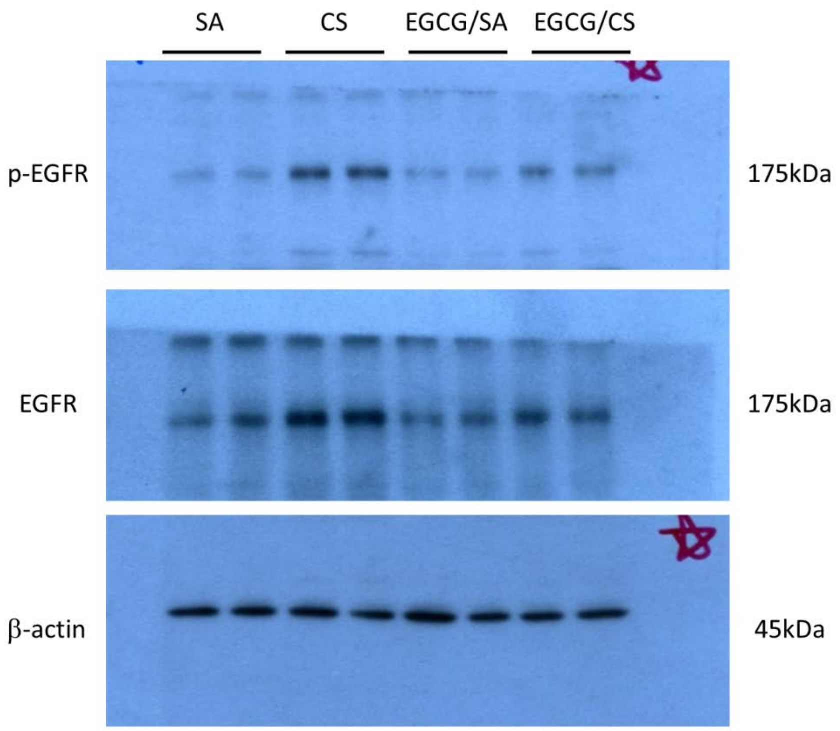

Figure S1. Full images of Western blot.

EGFR, Epidermal growth factor receptor.
